# Modeling and Preventing Progressive Hearing Loss in Usher Syndrome III

**DOI:** 10.1038/s41598-017-13620-9

**Published:** 2017-10-18

**Authors:** Ruishuang Geng, Akil Omar, Suhasini R. Gopal, Daniel H.-C. Chen, Ruben Stepanyan, Martin L. Basch, Astra Dinculescu, David N. Furness, David Saperstein, William Hauswirth, Lawrence R. Lustig, Kumar N. Alagramam

**Affiliations:** 1 Department of Otolaryngology-Head and Neck Surgery, University Hospitals Cleveland Medical Center, Case Western Reserve University, Cleveland, Ohio, 44016 USA; 2 Department of Otolaryngology-Head and Neck Surgery, University of California San Francisco, San Francisco, CA 94143 USA; 3 Department of Ophthalmology, University of Florida, Gainesville, FL 32610 USA; 4 School of Life Sciences, Keele University, Keele, Staffordshire, ST5 5BG UK; 5 Vitreoretinal Associates of Washington, Seattle, WA 98104 USA; 6 Department of Otolaryngology-Head and Neck Surgery, Columbia University, New York, NY 10032 USA; 7 Genetics and Genome Sciences, Case Western Reserve University, Cleveland, Ohio 44016 USA; 8 Neurosciences, Case Western Reserve University, Cleveland, Ohio 44016 USA

## Abstract

Usher syndrome type III (USH3) characterized by progressive loss of vision and hearing is caused by mutations in the clarin-1 gene (*CLRN1*). *Clrn1* knockout (KO) mice develop hair cell defects by postnatal day 2 (P2) and are deaf by P21-P25. Early onset profound hearing loss in KO mice and lack of information about the cochlear cell type that requires *Clrn1* expression pose challenges to therapeutic investigation. We generated KO mice harboring a transgene, TgAC1, consisting of *Clrn1*-UTR (*Clrn1* cDNA including its 5′ and 3′ UTR) under the control of regulatory elements (*Atoh1* 3′ enhancer/β-globin basal promoter) to direct expression of *Clrn1* in hair cells during development and down regulate it postnatally. The KO-TgAC1 mice displayed delayed onset progressive hearing loss associated with deterioration of the hair bundle structure, leading to the hypothesis that hair cell expression of *Clrn1* is essential for postnatal preservation of hair cell structure and hearing. Consistent with that hypothesis, perinatal transfection of hair cells in KO-TgAC1 mice with a single injection of AAV-*Clrn1*-UTR vector showed correlative preservation of the hair bundle structure and hearing through adult life. Further, the efficacy of AAV-*Clrn1* vector was significantly attenuated, revealing the potential importance of UTR in gene therapy.

## Introduction

Hereditary hearing loss (HHL) is a common sensory deficit often associated with hair cell defects^1^, and a good example of that is hearing loss linked to Usher syndrome (USH). USH is an autosomal recessive disorder and a common cause of combined deafness and blindness^2^. One subtype, USH3, is characterized by post-lingual, progressive hearing loss (PHL) and loss of vision with or without vestibular dysfunction^3–5^. The age of detection of hearing loss in USH3 subjects ranges from infancy to greater than 35 years, with most cases being diagnosed by the age of 10 years^4,5^. This provides an important therapeutic window before the onset of symptoms. However, lack of a progressive hearing loss model for USH3 has stymied progress in this regard.

USH3 is caused by mutations in the clarin-1 (*CLRN1*) gene, which encodes a four-transmembrane protein (CLRN1) closely related to tetraspanins^6,7^. *CLRN1* ^*Y176X*^ and *CLRN1* ^*N48K*^ are the most common mutations among USH3 cases^4,6,7^. Mouse knockout mutants generated to mimic nonsense (*Clrn1* ^*KO*/*KO*^; referred to as “KO”) or missense (*Clrn1* ^*N48K*/*N48K*^) mutations in humans are deaf by P25^8–10^. Zebrafish *clrn1* ^*KO/KO*^ larvae are hearing impaired as well^11^. Hair cell mechanotransduction is attenuated in mice and zebrafish clarin-1 mutants^8,10,11^. Further, human CLRN1 protein, expressed in mouse or zebrafish hair cells, localizes to the hair bundle; whereas, human CLRN1^N48K^ fails to reach the hair bundle and is largely retained in the endoplasmic reticulum^10,11^. Studies in clarin-1 mutant mice and zebrafish point to defects in the mechanosensory hair cells as the causative factor for inner ear dysfunction associated with clarin-1 mutation. The data discussed above strongly suggest clarin-1 is an essential hair cell protein across species.

The mechanosensory structure of the hair cell is the stereocilia (“hair”) bundle located on the apical surface of the cell. Deterioration of hair bundle morphology is observed as early as P2 in *Clrn1* mutant mice^10^, making them a poor mammalian model to investigate postnatal therapeutic intervention, since treatment would have to be instituted prior to this damage. Additional challenges include lack of information regarding the cochlear cell type(s) that require *Clrn1* expression and the timing of expression. Previous work showed that mouse *Clrn1* mRNA is expressed constitutively in the inner ear (based on RT-PCR analysis), and mRNA *in situ* hybridization (ISH) of embryonic and neonate inner ear shows that *Clrn1* is expressed in the spiral ganglion neurons (SGN) and hair cells (HCs)^7,8^.

The primary amino acid sequence of the clarin-1 protein (CLRN1) predicts 4-transmembrane domains similar to the tetraspanins family of membrane proteins^7^. Members of this family are considered structural proteins that interact laterally with other membrane proteins such as ion channels, integrins, and other tetraspanins to form tetraspanin-enriched microdomains^12,13^. Tetraspanins have been implicated in a variety of cellular functions, including regulation of cholesterol-dependent microdomains with actin cytoskeleton^12,14^. *In vitro* studies show that CLRN1 forms membranous cholesterol-rich compartments on plasma membranes, and it interacts with and regulates the machinery involved in actin filament organization^15^. Proteomics analyses revealed a number of clarin-1-interacting proteins involved in a variety of cellular function, including regulation of the actin cytoskeleton^15^. Harmonin, the Usher type IC gene product, interacts with many Usher proteins^16–18^. It is reported to interact directly with F-actin *in vitro* and stabilize F-actin when it is expressed in HeLa cells^19^. We propose that CLRN1 is necessary for maintenance of the F-actin core of the hair cell stereocilia and it mediates this function by interacting with other proteins involved in the regulation of actin cytoskeleton.

Based on clarin-1 literature discussed earlier, we predict that down regulation of CLRN1 expression in hair cells will result in the progressive disintegration of bundle structure and hearing loss; conversely, preservation of hearing in a clarin-1 mutant will require restoration of wild-type clarin-1 expression in hair cells in the mutant background. Here, we test our predictions and address several critical questions relating to the role of *Clrn1* in USH3: (1) Is the expression of *Clrn1* in SGNs and HCs necessary for the development of hearing, and is hearing (preservation) dependent on postnatal expression of *Clrn1*? (2) What is the consequence of postnatal down regulation of *Clrn1* expression to hair cells? (3) Can a USH3 mouse model that displays delayed onset PHL be developed? (4) If so, is it possible to block or constrain PHL and prevent deafness in this model using viral gene therapy? (5) What is the importance of the untranslated region (UTR) of the *Clrn1* mRNA with regard to gene therapy? Answers to these questions are critical to understanding the role of *Clrn1* in the ear and in guiding future efforts to develop viable therapies for USH3 patients.

To address these questions, we generated KO mice carrying a transgene, TgAC1, that consisted of *Clrn1*-UTR (*Clrn1* cDNA flanked by 5′ and 3′ UTR) under the control of regulatory elements (*Atoh1* 3′ enhancer/β-globin basal) known to direct expression of the target gene in hair cells during development and significantly down regulate it after birth (<P10)^20^. These mice are designated ‘KO-TgAC1’. We also designed AAV vectors that carry the coding sequence of *Clrn1*, with or without the UTR, and injected them in the ears of KO or KO-TgAC1 mice at postnatal day 2–3 (P2-P3). The KO-TgAC1 mice develop hearing, with ABR thresholds comparable to wild-type mice at a young age (P22), but fail to maintain that function over time. Our results suggests hair cell expression of *Clrn1* is sufficient for hearing development, and postnatal hearing (preservation) is dependent on hair cell expression of *Clrn1*. Consistent with the latter observation perinatal administration of AAV-*Clrn1*-UTR vector blocks that progression of hearing loss in the new (KO-TgAC1) USH3 mouse model. The AAV based preservation of hearing in the new USH3 model (KO-TgAC1 mice) also provide proof of concept that gene therapy is effective in mitigating sensory deficit associated with *CLRN1* mutation.

## Results

### Conditional expression of *Clrn1* in hair cells in the KO background

**Figure 1:**
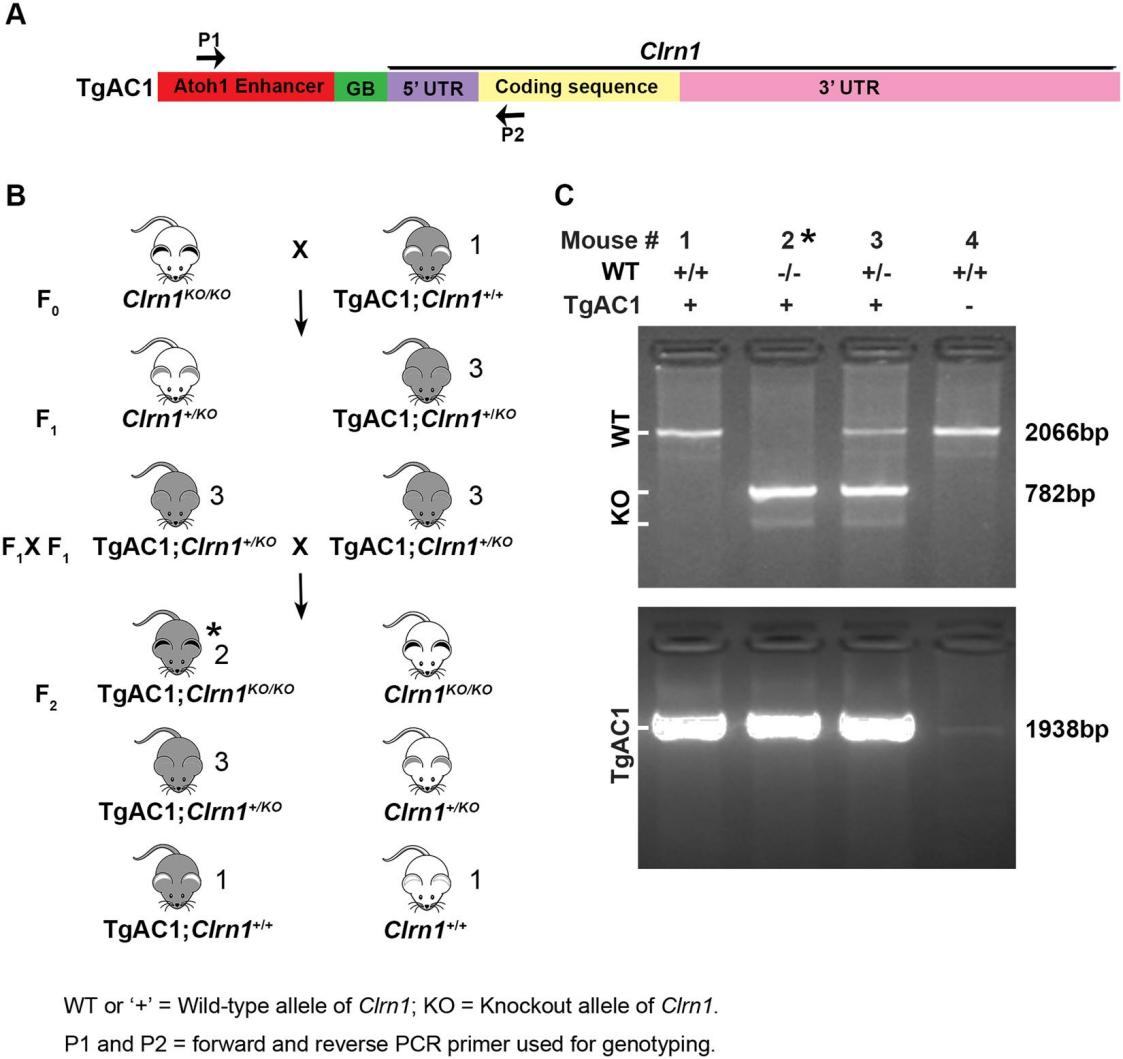
Generation of KO-TgAC1 mice. (**A**) A schematic of a transgene construct. The transgene construct is composed of an *Atoh1* enhancer sequence fused to the beta globin basal (GB) promoter sequence. The *Clrn1* cDNA fused downstream of the *Atoh1* regulatory elements is composed of the 5′ UTR sequence, the coding sequence (isoform 2) and the 3′ UTR sequence. (**B**) The breeding scheme used to generate KO-TgAC1 mice. The expanded symbol for the KO-TgAC1 mice is “TgAC1; *Clrn1* ^*KO*/*KO*^”, marked by an asterisk in the F2 generation. The number next to the mouse corresponds to the lane number in panel C. (**C**) PCR-based genotyping to identify the WT (2066 bp), KO (782 bp) and TgAC1 (1938 bp) allele of *Clrn1*. *F2 mice with the desired genotype, TgAC1; *Clrn1* ^*KO*/*KO*^.

**Figure 2:**
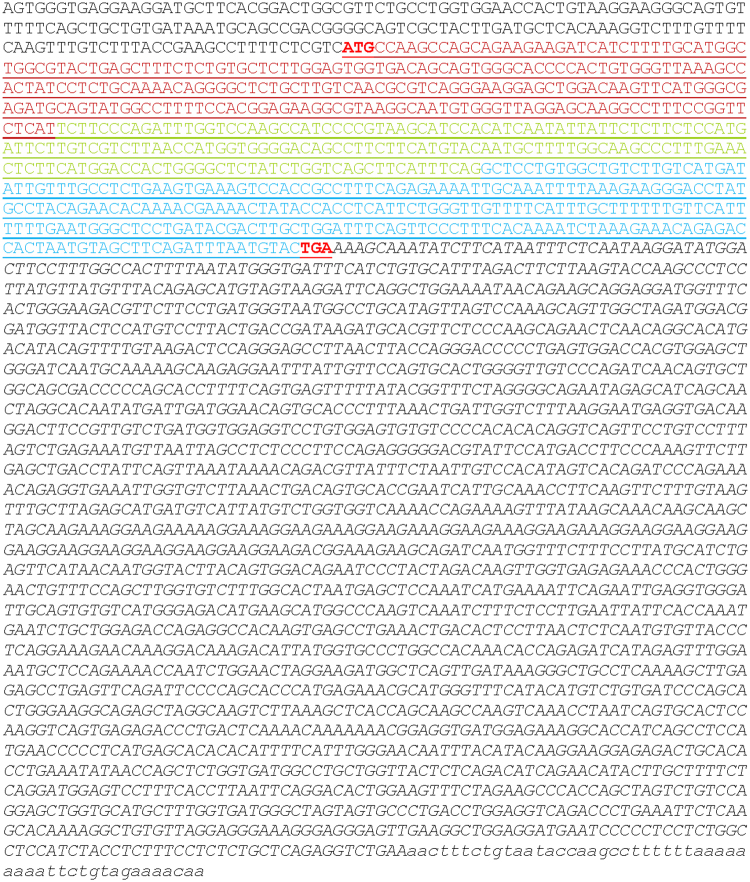
Mouse clarin-1 transcript. 5′ UTR, 172 bp (black font); coding sequence, 696 bp (underlined) start ‘ATG’ and stop ‘TGA’ codon indicated (bold, red font); exon 1 (red font), exon 3 (green font) and exon 4 (blue font); 3′UTR, 2158 bp (black font, italicized text; lower case italicized text indicates additional bases found in the GenBank sequence (Accession number: NM_153384.3) not included in the TgAC1 construct.

The scheme to generate and confirm the genotype of the transgenic mice is shown in Fig. 1. Mice homozygous for the KO allele (*Clrn1* ^*KO*/*KO*^) and carrying the transgene are designated “KO-TgAC1” (Fig. 1). *Atoh1* enhancer-mediated expression is detectable in mouse cochlear hair cells from embryonic stages to a week after birth^20,21^. The transgene (TgAC1) consists of the *Clrn1* cDNA sequence with the UTR sequence fused downstream of the *Atoh1* enhancer and β-globin basal promoter sequence (Fig. 1A). Translated and untranslated regions of the *Clrn1* cDNA sequence included in the TgAC1 construct are depicted in Fig. 2.

**Figure 3:**
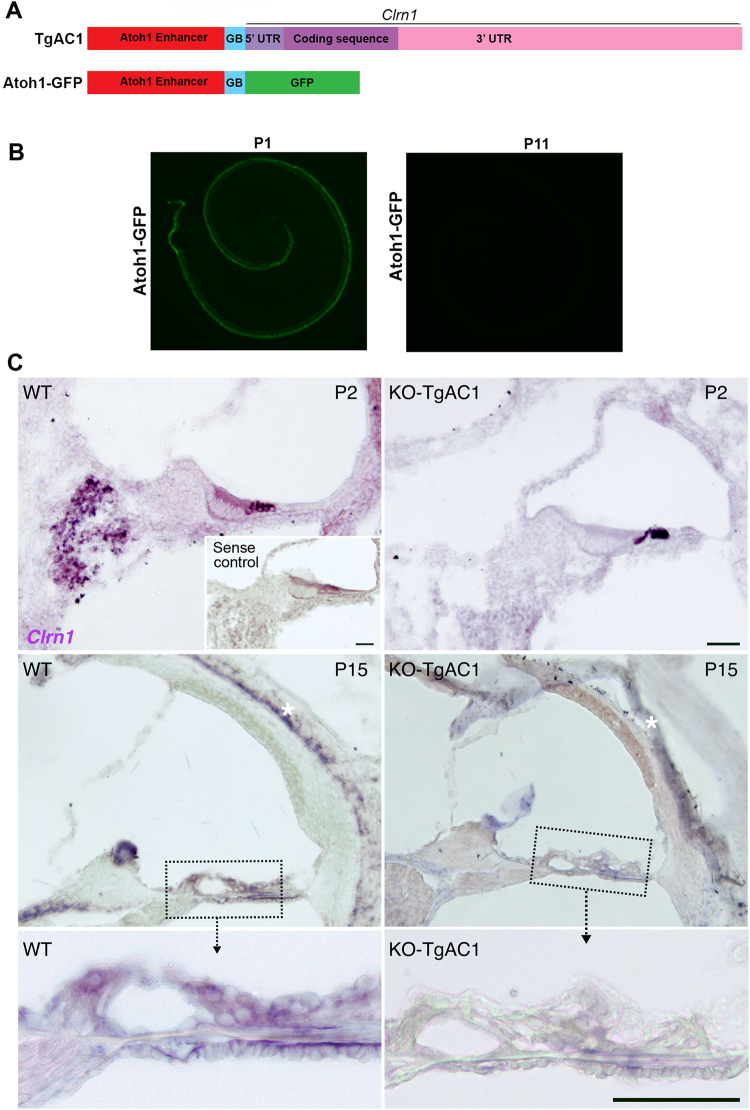
Conditional expression of *Clrn1* in KO-TgAC1 mice. (**A**) The schematic diagram shows *Atoh1*-*Clrn1* and *Atoh1*-*GFP* transgene constructs. (**B**) *GFP* expression in the organ of Corti at P1 and P11 from the *Atoh1*-*GFP* transgenic line. (**C**) *Clrn1* ISH on cochlear duct sections from WT or KO-TgAC1 mice at P2 (top row) and P15 (second and bottom row). The inset (sense probe, top row) and asterisks (labyrinth cartilage and tectorial membrane, second row) show background staining. Scale bar = 50 µM.

The *Atoh1* regulatory element (*Atoh1* 3′ enhancer/β-globin promoter/GFP) has been shown to be sufficient to direct expression of the target gene in hair cells of the organ of Corti during development and a few days after birth^20,22^. None of the antibodies to mouse CLRN1 tested was specific in immunohistochemistry. Therefore, we used alternate approaches to visualize control of the *Atoh1* 3′ regulatory enhancer in the organ of Corti. A mouse harboring an *Atoh1*-*GFP* transgene (Fig. 3A) served as a control to monitor conditional expression of the target protein in the organ of Corti. The GFP expression was robust in the inner hair cells (IHCs) and outer hair cells (OHCs) from the apex to the base of the cochlea at P1 but undetectable by P11 (Fig. 3B). The GFP expression data implies that, beyond P11, CLRN1 expression in cochlear hair cells of the TgAC1-KO mice will be significantly down regulated or absent after P11. The expression of *Clrn1* mRNA in the cochlear duct sections from WT and KO-TgAC1 mice at P2 and P15 were examined by ISH. At P2, *Clrn1* is expressed in IHCs and OHCs and the ganglion cells in the WT (Fig. 3C), consistent with earlier reports^7,8^. In the KO-TgAC1 mice, *Clrn1* expression is restricted to the hair cells (Fig. 3C). At P15, *Clrn1* expression persists in the WT hair cells but not in the KO-TgAC1 hair cells (Fig. 3C middle and bottom row). ISH results show temporal and spatial regulation of *Clrn1* expression in the cochlea of KO-TgAC1 mice, and these results match expectations for gene expression under the control of the *Atoh1* 3′ enhancer element^20,22^. We hypothesized that postnatal down regulation of *Clrn1* expression in the hair cells will result in progressive loss of hearing in mice.

#### KO-TgAC1 mice display delayed onset PHL

**Figure 4:**
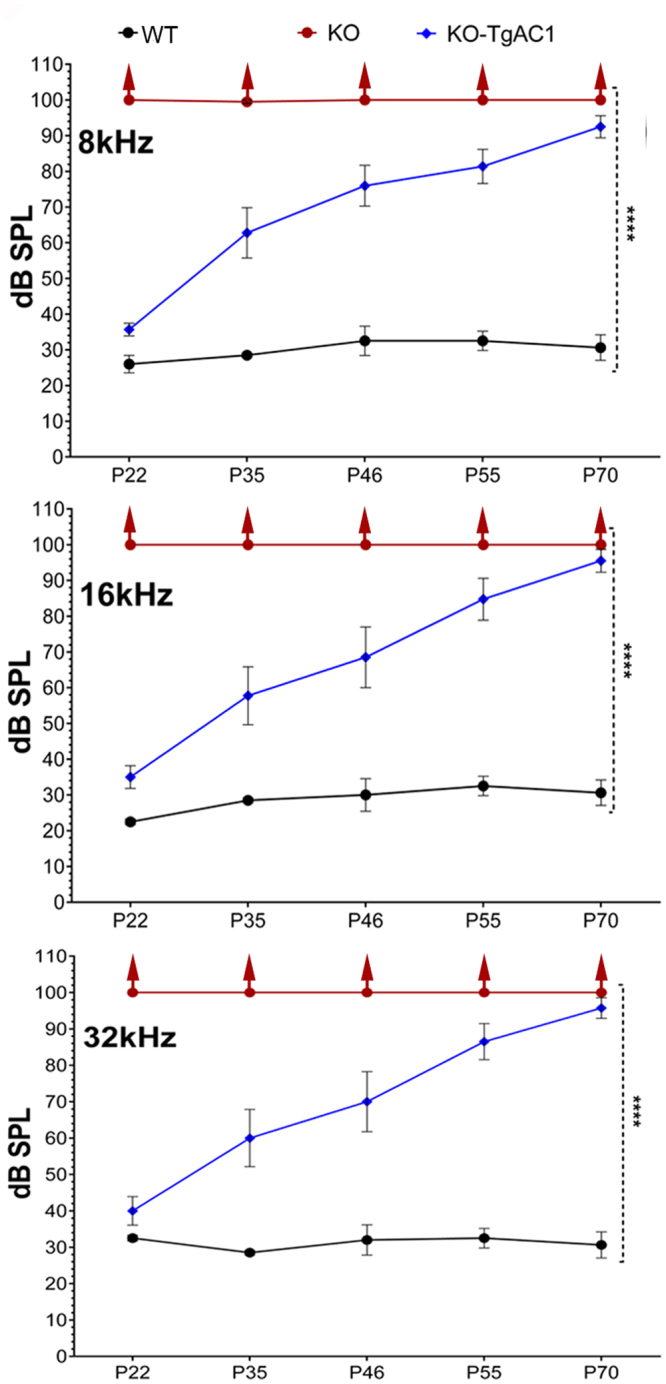
Progressive hearing loss in *Clrn1* KO mice harboring the TgAC1 transgene. To monitor hearing over time, ABR thresholds were recorded longitudinally from the same set of mice in each group (genotype). ABR thresholds of WT (n = 5), KO (n = 10) and KO-TgAC1 (n = 10) mice from P22 to P70 at 8, 16 and 32 kHz. ****p < 0.0001. No ABR was generated in the KO mice at the highest intensity (100 dB SPL) tested; the upward arrow indicates that the threshold is greater than 100 db SPL. The error bar in each panel represents standard error of the mean.

Hearing in the KO-TgAC1, KO and wild-type (WT) control mice, was monitored from P22 to P70 using auditory evoked brainstem response (ABR) thresholds at 8, 16 and 32 kHz (Fig. 4). The median ABR threshold in the WT mice was 30 ± 5 dB SPL. At P22, the KO mice showed profound hearing loss while most of the KO-TgAC1 mice had ABR thresholds closer to the WT mice at all frequencies tested. Over the next 40 days, hearing sensitivity in the KO-TgAC1 mice gradually decreased, reaching a state of profound hearing loss by P70 (ABR threshold >85–90 dB SPL). One-way ANOVA confirmed the statistical significance of these results (*p* ≤ 0.0001). Our results suggest: (1) hair cell expression of *Clrn1* is necessary to develop hearing; (2) postnatal expression of *Clrn1* in hair cells is necessary to maintain hearing after birth; and (3) compared to KO mice, the KO-TgAC1 mice display delayed onset progressive hearing loss.

### Hair cell-specific expression of *Clrn1* rescues core phenotype in the KO mice

**Figure 5:**
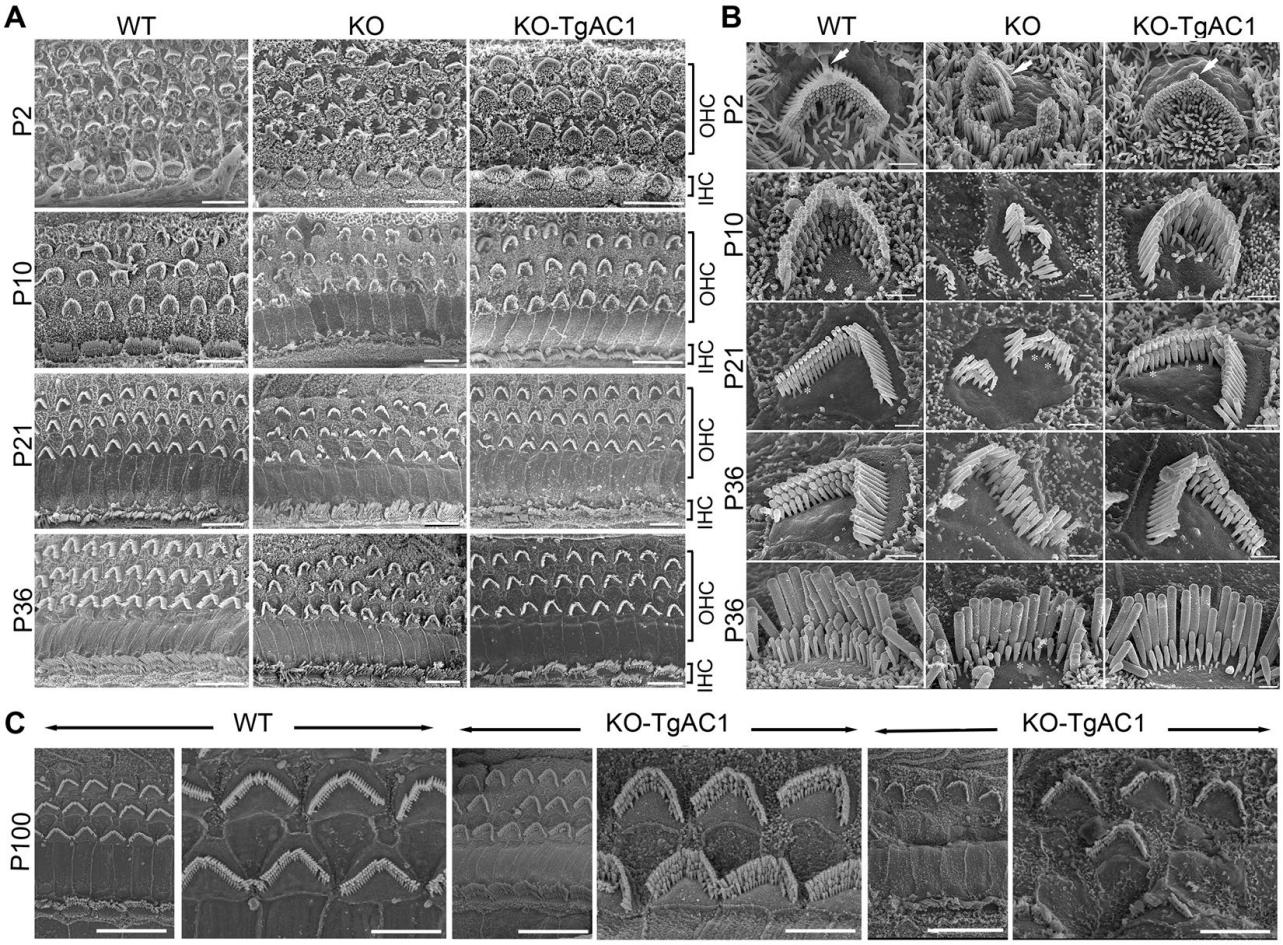
Delayed onset of hair cell damage in KO-TgAC1. (**A**) FESEM images of WT, KO and KO-TgAC1 organ of Corti from mid-cochlear regions. At all ages illustrated, the hair bundle morphology appears disrupted in all rows of the OHCs in the KO, whereas the hair bundle morphology in the KO-TgAC1 mice is grossly comparable to the WT specimen. Scale bars = 10 μm. (**B**) At higher magnification, disturbances of the hair bundles apparent in the KO specimens are much reduced in the KO-TgAC1 specimens. The upper four rows show OHCs at P2-P36. The KO shows disturbed bundles with splits in the rows and missing stereocilia, and distorted apical surfaces, while the KO-TgAC1 has a near normal appearance. Some of the short stereocilia in all three mice are missing (*), an effect which is greater in KO-TgAC1 and greater still in the KO compared with the WT. The bottom row shows IHCs at P36. In both the KO and KO-TgAC1 mice, short stereocilia are missing compared with the WT, but overall the hair bundle in KO-TgAC1 mice is more similar to the WT than the KO. Scale bars = 1 μm. (**C**) At P100, the majority of the OHCs along the cochlear turn are severely affected (panels to the right) with a few patches of the organ of Corti that appear to be less severely affected (panels to the center) compared to the WT (panels to the left). The organ of Corti from the mid-basal turn of the cochlea is shown here. All mice are maintained in the C57BL/6J background; five mice (n = 5) per each genotype were used for the SEM analysis. Scale bars = 5 μm.

To investigate the structural correlate associated with delayed onset progressive hearing loss in the KO-TgAC1 mice, hair bundle morphology was evaluated using field emission scanning electron microscopy (FESEM) of the mid-cochlear regions of WT, KO and KO-TgAC1 mice at P2, P10, P21, P36 and P100. At relatively low magnification, compared to WT mice, bundle morphology in all hair cells in KO mice was disrupted at all ages examined (Fig. 5A). The disarray was more prominent in the outer hair cells (OHCs) than in the inner hair cells (IHCs). Virtually all the OHC bundles appeared disturbed to some degree. In the KO-TgAC1 mice, the OHC and IHC bundles maintained relatively normal morphology (Fig. 5A). A higher magnification examination primarily of the OHC bundles was also undertaken. In contrast to the KO mice, the appearance of the bundle and shape of the cuticular plate in the KO-TgAC1 mice was grossly comparable to the WT mice from P2 to P36 (Fig. 5B). However, KO-TgAC1 mice begin to show signs of degeneration as early as P21; the shortest row of stereocilia became depleted to a greater extent than the age-matched WT mice (Fig. 5B). The IHC bundle morphology was similar in both the KO and KO-TgAC1 mice and showed loss of the shortest one to two rows of stereocilia with age. At P36, loss of the shortest row of stereocilia in the IHC is apparent (Fig. 5B). After P30, the bundle structure in IHCs and OHCs in the KO-TgAC1 mice deteriorated progressively over time, although disruption in bundle structure in the OHCs was readily identifiable or more prominent compared to IHCs. By P100, some ears had patches in which only a few OHC and IHC bundles remained, and these were severely disrupted (Fig. 5C). These studies show that the hair bundle phenotype in the KO-TgAC1 mice is consistent with the delayed onset, progressive hearing loss observed in this new model of USH3, suggesting that sustained postnatal expression of *Clrn1* in hair cells is required to maintain hair bundle structure and hearing in adult mice.

### AAV2 or AAV8 transfect cochlear hair cells *in vivo*

**Figure 6:**
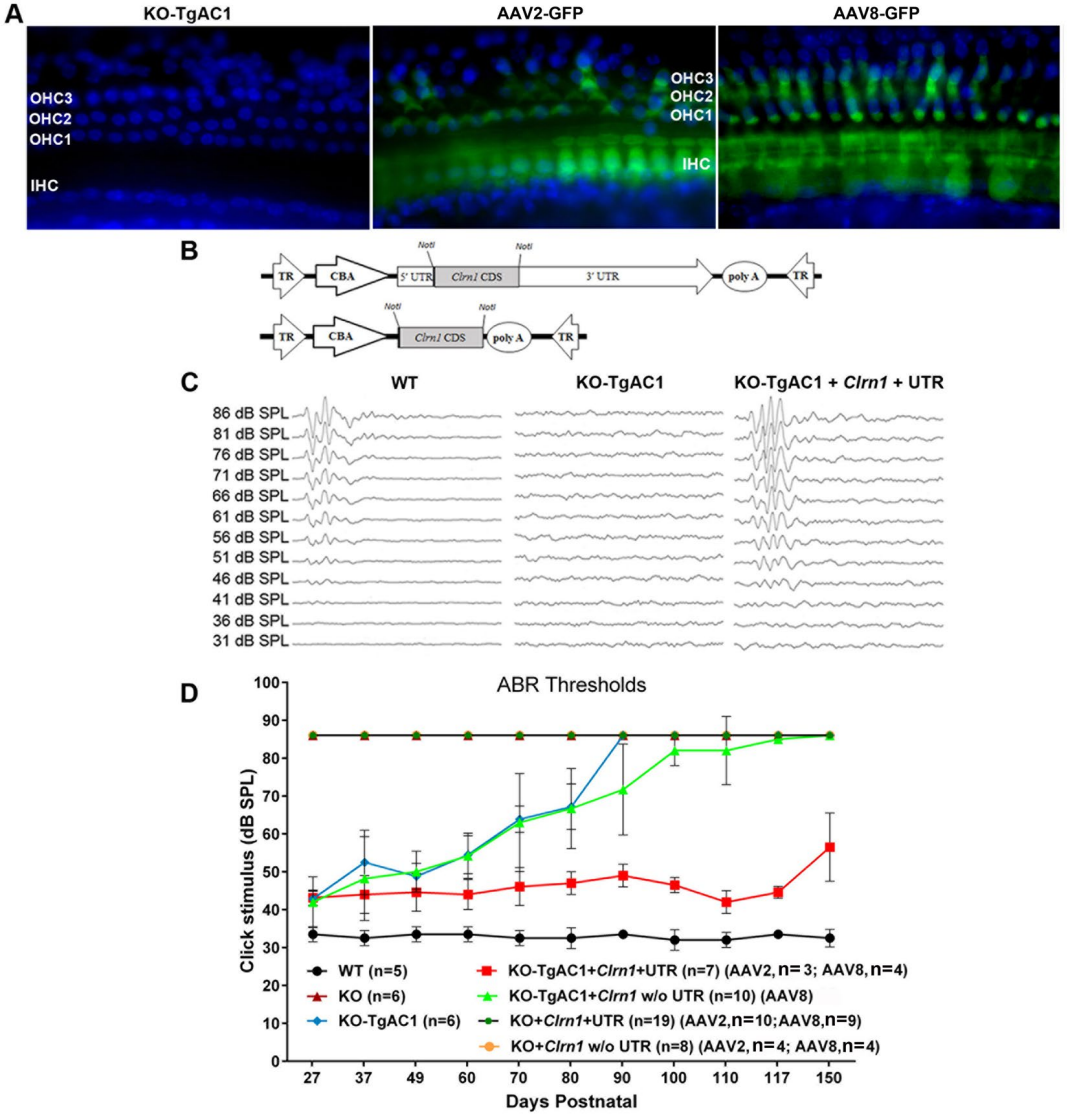
AAV-mediated gene therapy in the KO-TgAC1 USH3 model. (**A**) Surface preparation of the organ of Corti from KO-TgAC1 mice stained with DAPI (left panel), transfected with AAV2 -GFP (middle panel) or AAV8-GFP (right panel). AAV2-GFP and AAV8-GFP were used to assess viral delivery to the cochlea of KO-TgAC1. High titer stock (>10^13^ vg/ml) of AAV2 or 8-GFP was injected through the RWM of the KO-TgAC1 mice at P1-P3, and the organ of Corti was examined for GFP expression in the cochlea whole mount at P10 by immunofluorescence using anti-GFP antibody. Almost all IHCs were GFP positive (green), whereas a mosaic pattern of GFP positive cells was observed in the three rows of OHCs. Representative specimens from the mid-basal turn of the cochlea from mice injected with AAV2-GFP or AAV8-GFP  are shown in the middle and right panels. (**B**–**D**) Hearing restoration in the KO-TgAC1 mice. (**B**) Schematic representation of AAV vector constructs used in gene rescue experiments. The AAV expression cassette contains the mouse clarin-1 coding sequence (CDS) with or without its UTR sequences. Both constructs were packaged in either serotype 2 or serotype 8 AAV capsids. The *Clrn1* cDNA expression in each vector is driven by the ubiquitous small CBA promoter. The expression cassette is flanked by inverted terminal repeats (TR) of AAV serotype 2; poly-A, SV40 polyadenylation signal. (**C**) Representative ABR traces in response to click stimulus from WT, KO-TgAC1 and rescued KO-TgAC1 mice following delivery of 2 μl AAV2 or AAV8-*Clrn1*-UTR construct. At P100, waveforms from WT and KO-TgAC1 mice injected with AAV2-*Clrn1*-UTR appear normal and similar, while non-injected KO-TgAC1 mice show no ABR responses at the sound levels tested. (**D**) AAV2 or AAV8 containing the *Clrn1* gene, with or without the UTR sequence, were directly injected through the RWM of the KO or KO-TgAC1 mice at P1-P3 when the hair cells in KO-TgAC1 mice are comparable to the WT hair cells. To monitor preservation or loss of hearing over time, ABR thresholds were recorded from the same set of mice in each group (genotype). ABRs evoked with click stimulus recorded longitudinally from the same mice 4 to 22 weeks after birth (P27 to P150). The KO-TgAC1 mice injected with AAV2 or AAV8-*Clrn1*-UTR (n = 7) (red line) showed significant preservation of hearing through their adult life compared to the KO-TgAC1 mice injected with *Clrn1* without UTR (n = 10) or non-injected sham surgery KO-TgAC1 (n = 6). There was no statistically significant differences in ABR thresholds over time for the same construct delivered with either AAV2 or AAV8, thus permitting the grouping of results from AAV2 and AAV8. These groupings are indicated within paranthesis in the key to the legend, where the second numeral after AAV serotype is the n value.

We next sought to determine whether viral-mediated gene therapy could prevent or further delay the onset of hearing loss in the KO-TgAC1 model. For these studies, constructs carrying the WT *Clrn1* cDNA with or without the sequences representing the 5′ and 3′ UTRs were delivered to the cochlea of the KO-TgAC1 mice at P1-P3 using adeno-associated virus (AAV) serotype 2 or 8 through the round window membrane (RWM) as described previously^23^. To assess the extent of transfection of the organ of Corti by AAV, the WT mice were initially injected with AAV2-GFP and examined at P10. GFP was detected in almost all IHCs, whereas a mosaic pattern of expression was observed among the three rows of OHCs. Representative specimens from the mid-basal turn of the cochlea from two of the five mice injected with AAV2-GFP and AAV8-GFP are shown in Fig. 6A. As reported previously, AAV2 or AAV8 were much more effective at transfecting IHCs than OHCs^23,24^, though the precise reason behind this variable transfection in hair cells remains unknown.

### AAV-*Clrn1*-UTR vectors constrain PHL in the KO-TgAC1 mice

AAV2 or AAV8 containing *Clrn1* cDNA, with or without the UTR (Fig. 6B), were injected through the RWM of the KO or KO-TgAC1 mice at P1-P3, an age at which the hair cells in the KO-TgAC1 mice are comparable to those in the WT. In these mice, ABRs were recorded from 4 to 22 weeks after birth (P27 to P150) to monitor hearing preservation longitudinally. Seven KO-TgAC1 mice received AAV-*Clrn1*-UTR (KO-TgAC1-AAV-*Clrn1*-UTR) at P1-P3, and longitudinal ABR recordings were pursued until P150 (Fig. 6D). These mice showed sustained hearing preservation from the start (Fig. 6D). The suppression of progressive hearing loss by gene therapy is evident earlier on in the treated mice compared to the untreated (KO-TgAC1 subject to sham surgery) mice (n = 6, Fig. 6D). Starting at P70, the suppression was statistically significant for KO-TgAC1-AAV-*Clrn1*-UTR compared to KO-TgAC1 (Student’s *t*-test, *p* ≤ 0.02). However, the effect of gene therapy in KO-TgAC1 mice treated with AAV-*Clrn1*-UTR is outstanding starting at P90, when all other mice progress to profound hearing loss status (Fig. 6D). From P90 to P150, the mean difference in the ABR threshold between the KO-TgAC1 and KO-TgAC1-*Clrn1*-UTR was 38.3 dB SPL, which translates to improved hearing by more than three orders of magnitude. Our analysis shows that the threshold difference between the KO-TgAC1 and KO-TgAC1-*Clrn1*-UTR groups is statistically significant at 95% power (*p* < 0.01). In contrast, from P90 to P150, the mean difference in ABR threshold between KO-TgAC1 mice treated with AAV-*Clrn1*-without UTR (n = 10) and untreated KO-TgAC1 mice (n = 6) (Fig. 6D) is ≈5 dB SPL, and the difference is not statistically significant. These results demonstrate that AAV-mediated gene therapy can prevent deafness in the USH3 background, and AAV-*Clrn1*-UTR is the causal variant associated with the potent/stable therapeutic effect.

### AAV-*Clrn1*-UTR vectors preserve the mechanosensory structure of hair cells

**Figure 7:**
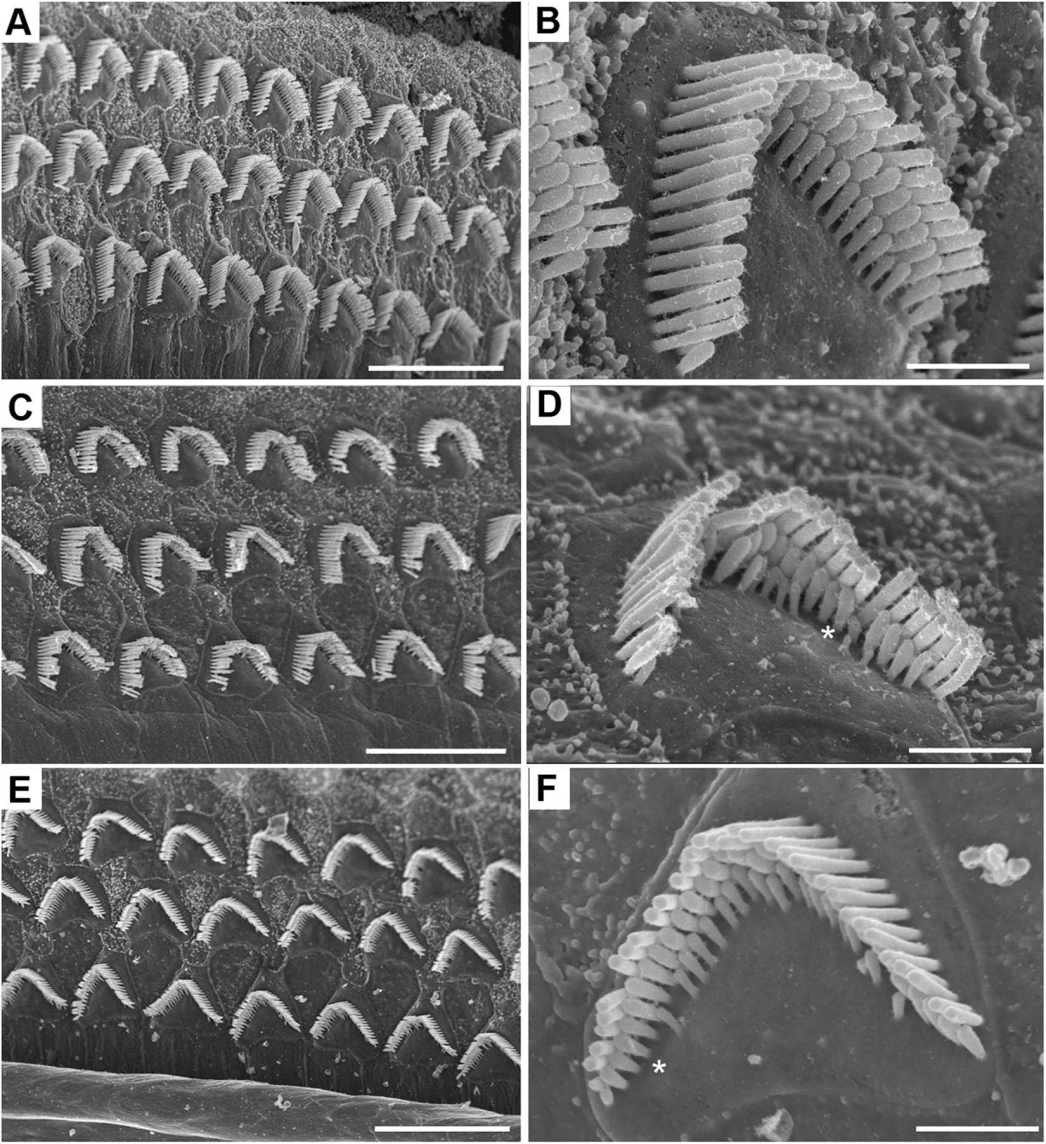
FESEM images of WT and AAV2-*Clrn1*-UTR injected KO-TgAC1 mice at P100. All images are from the sub-apical region of the cochlea. WT shows normal OHC bundles (**A**,**B**). The injected KO-TgAC1 mouse with 65 dB SPL ABR threshold (to click stimulus) showed more spread-out or disorderly hair bundles in general (**C**) compared to the injected KO-TgAC1 mouse with 40 dB SPL ABR threshold (to click stimulus) (**E**). However, in the closeups of the OHC bundles, some short stereocilia are missing in both examples (**D**,**F** *). Scale bars (**A**,**C**,**E**) = 10 µm; (**B**,**D**,**F**) = 1 µm.

To determine the anatomical correlates of hearing preservation in KO-TgAC1 mice following AAV8-*Clrn1*-UTR injection, we compared hair bundle morphology from mice that displayed moderate hearing preservation (65 dB SPL ABR threshold) and good preservation (40 dB SPL ABR threshold) to WT mice at P100. The hair bundle morphology was significantly better preserved in the transfected KO-TgAC1 mice (Fig. 7) compared to the WT control (Fig. 7A,B). However, the integrity of the bundle morphology was commensurate with the degree of hearing preservation in the injected mice (Fig. 7C,D,E,F). Thus, the rescue of the hair cell bundle phenotype closely correlates with the rescue of hearing in the injected KO-TgAC1 mice. However, ABR thresholds did not reach normal threshold levels in injected KO-TgAC1 mice. We believe this is due to the variable transfection of OHCs, leading to variability in hearing thresholds. Nevertheless, these data demonstrate that virally mediated gene transfer can provide robust, long-term preservation of hearing in the delayed onset hearing loss model of USH3.

### Immunolocalization of CLRN1-HA in IHCs and OHCs following AAV transfection

**Figure 8:**
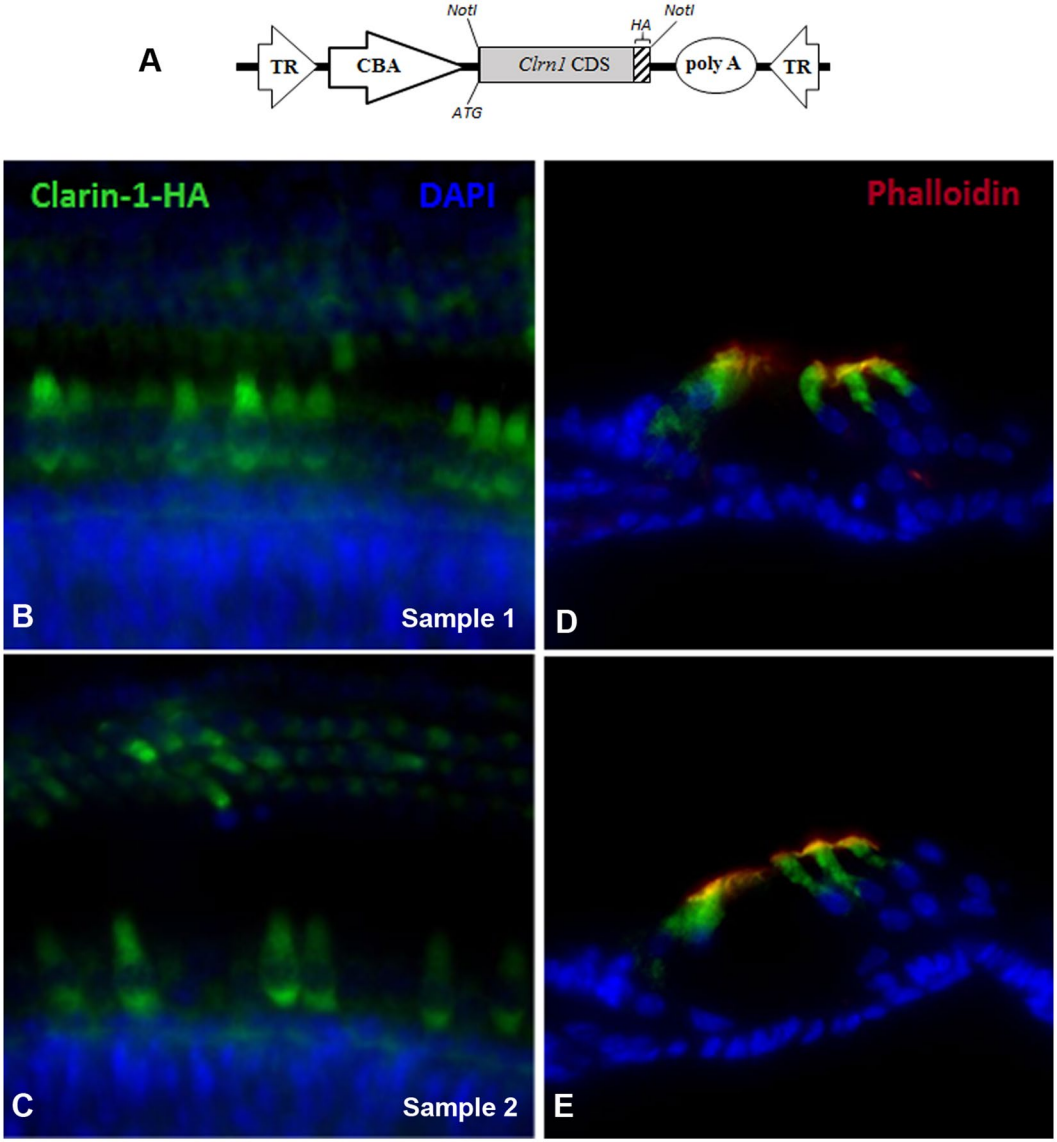
AAV-mediated *Clrn1*-HA gene expression in hair cells of the organ of Corti in wild-type mice. High titer stock (>10^13^ vg/ml) of AAV8-*Clrn1*-HA was injected through the RWM of the wild-type mice at P1-P3, and the organ of Corti was examined for HA expression in the cochlea whole mount at P10 by immunofluorescence using anti-HA antibody.** (A**) Schematic representation of the AAV vector construct used for immunolocalization. The AAV expression cassette contains the mouse *Clrn1* coding sequence (CDS), without its UTR sequences, fused to the CDS for HA epitope. Serotype 8 AAV capsid was used to package this construct. The ubiquitous small CBA promoter drives expression of *Clrn1*-*HA* in the vector. The expression cassette is flanked by inverted terminal repeats (TR) of AAV serotype 2; poly-A, SV40 polyadenylation signal. Immunolocalization of CLRN1-HA in the organ of Corti of transfected mice using antibodies to the HA epitope. Representative specimens from the mid-basal turn of the cochlea from 2 of the 5 mice injected with AAV8-*Clrn1*-HA are shown in the left top (**B**) and bottom (**C**) panels. Cross sections of the cochlea were examined to determine whether the AAV8-mediated transfection was restricted to the hair cells (panels D and E).

Immunolocalization of CLRN1 in the organ of Corti following AAV transfection would be ideal for the interpretation of the gene therapy data. However, specific labeling could not be obtained using the reported antibodies to the mouse CLRN1^25^, or the antibodies generated by us to the mouse CLRN1 or to the zebrafish Clrn1 antibodies (Novus Biologicals). Alternatively, we used viral vectors carrying *Clrn1* fused to HA (hemagglutinin) epitope (AAV8-*mClrn1*-HA). Immunolocalization using antibodies to HA epitope from independent specimens showed transfection of most IHCs and a mosaic pattern of transfection of OHCs in the three rows (Fig. 8A–D); cross sections revealed that hair cells, not supporting cells, express CLRN1-HA (Fig. 8C,D). Data shown in Fig. 8, combined with results described earlier (Figs 3C and 4), are consistent with the idea that hearing preservation in AAV-*Clrn1* transfected TgAC1-KO mice is associated with hair cell expression of the target protein.

## Discussion

We generated a new USH3 mouse model that displays delayed-onset progressive hearing loss. To our knowledge, this is the first progressive hearing loss model for USH3. We developed this model by taking advantage of a proven regulatory element, the *Atoh1* 3′ enhancer^20,21^, to drive transient expression of *Clrn1* in hair cells from the embryonic to perinatal stage and then down regulate after birth. We generated mice carrying the “*Atoh1* enhancer-basal promoter-*Clrn1* cDNA” transgene (TgAC1) in the KO background (KO-TgAC1). We hypothesized that the KO-TgAC1 mice would display delayed onset hearing loss, and the ensuing decrease in hearing sensitivity would be more gradual (relative to the phenotype evidenced by the KO mice), resulting in a progressive hearing loss model. ABR data (Fig. 4) were consistent with the hypothesis. Further, postnatal (P1-P3) transfection of hair cells in the KO-TgAC1 mice with a single injection of AAV-*Clrn1*-UTR vector showed preservation of the hair cell structure and hearing through adult life in the new model of USH3 (Fig. 6). Our results suggest that hair cell expression of *Clrn1* is necessary for the development and postnatal preservation of hearing.

The viral gene therapy experiment involved 71 mice belonging to 7 groups tested longitudinally at various time points (Fig. 6D). To minimize variations in experimental and/or recording conditions, mice from the seven groups were processed (treated or untreated) and hearing evaluated within the same timeframe. To test hearing preservation, ABR thresholds to broadband click stimulus (as opposed to using pure tone stimuli) were recorded for two reasons. First, this method is adequate to address the important question namely, is it possible to block or slow down PHL in this model by viral gene therapy? If the PHL is constrained, we expect to keep deafness (total loss of hearing) at bay or delayed beyond P90, since the untreated KO-TgAC1 mice go deaf by P ≤ 90 (Fig. 6D). Second, we are working with a larger number (>20) of mice per recording session/time point (Fig. 6D) compared to characterization of PHL in the KO-TgAC1 model (10 per recording session/time point) (Fig. 4). Recording ABR thresholds to the click stimulus is quicker compared to recording ARB thresholds at multiple frequencies for each mouse. Our results (Fig. 6D) show that it is possible to constrain the progression of hearing loss and prevent early onset deafness in the KO-TgAC1 model via viral gene therapy. However, hearing thresholds in the AAV-*Clrn1*-UTR injected KO-TgAC1 mice were shy of the wild-type ABR threshold by 20–30 dB SPL. While variable transfection of outer hair cells by AAV vectors is a prime suspect, the long-term consequences of *Clrn1* deficiency in the ganglion cells and the impact on hearing sensitivity require further investigation. We acknowledge that the ABR threshold response to broadband click stimuli is dominated by 4–16 kHz frequency responses with minimal contribution from the high frequency (>16 kHz) region of the mouse cochlea (example, Keller *et al*. 2011^26^). Additional AAV gene transfer experiments are necessary to evaluate how well *Clrn1* gene augmentation restores hearing in the KO-TgAC1 model in the 16–32 kHz region. Nevertheless, from P90 to P150, the mean difference in the ABR threshold between the KO-TgAC1 and KO-TgAC1-*Clrn1*-UTR mice translates to improved hearing by more than 1,000 times in the treated animals (Fig. 6D). These results provide proof of concept that gene augmentation could mitigate hearing loss in USH3 patients.

Immunolocalization of HA epitope expression in the IHCs and OHCs of the organ of Corti following AAV8-*Clrn1*-HA transfection (Fig. 8A–E) is consistent with the idea that hearing preservation in AAV-*Clrn1* transfected TgAC1-KO mice is due to rescue of *Clrn1* expression in hair cells. Additional experiments, namely ABR thresholds and immunofluorescence data from KO-TgAC1 mice transfected with AAV2- or AAV8-*Clrn1*-*HA* construct, are necessary to unequivocally demonstrate whether hearing preservation in AAV-*Clrn1* transfected TgAC1-KO mice is due to rescue of *Clrn1* expression in hair cells. The labeling pattern (Fig. 8B,C) is similar to the pattern observed with AAV2-GFP and AAV8-GFP (Fig. 6A), suggesting that AAV2 and AAV8 produce similar results in our hands. Though IHCs and OHCs were transfected by the AAV8-*Clrn1*-HA vector (Fig. 8A), the intensity of HA expression in hair cells appears to be lower and more variable between hair cells (Fig. 8B,C), compared to GFP signals in the AAV2-GFP or AAV8-GFP transfected organ of Corti (Fig. 6A). This difference is clear when GFP expression in the IHC rows in AAV2-GFP and AAV8-GFP (Fig. 6A) is compared to HA expression in the IHC row of AAV8-*Clrn1*-HA transfected cells (Fig. 8B,C). We believe the reduced/variable expression is due to lack of UTR of *Clrn1* in the AAV8-*Clrn1*-HA construct. We surmise that *Clrn1* UTR sequences increase stability of the transcript and efficiency of translation. Although these immunofluorescence images (Figs 6 and 8) were captured under similar settings, we note that our interpretations are not based on quantitative data. Rigorous quantitative analyses are required to test the hypothesis that *Clrn1* UTR sequences increase stability of the transcript and efficiency of translation. We will test this hypothesis in future experiments.

Our results show that gene therapy is a promising approach to preserve hearing in USH3 patients. The results show that in KO-TgAC1 mice transfected with the *Clrn1* coding sequence only, the rescue effect is barely perceptible or modest at best, whereas the inclusion of the UTR sequence improved hearing sensitivity by nearly four orders of magnitude in the KO-TgAC1 USH3 mouse model. In contrast, the KO mice injected with AAV2 or AAV8 -*Clrn1*-UTR demonstrated no hearing preservation (Fig. 6C). We suspect that lack of efficacy in the KO mice is because either AAV2 or AAV8 -*Clrn1*-UTR was injected after the onset of hair cell morphologic changes (P1-P3). This finding underscores the need to intervene with gene therapy prior to the onset of degeneration when treating a genetic defect that causes structural damage in the organ of Corti.

The results also emphasize that the UTR is critical for *Clrn1* gene therapy. Interestingly, the *Clrn1* 3′ UTR sequence (2.1 kb) is 3 times longer than the coding sequence (<0.7 kb) and 12 times longer than the 5′ UTR (0.17 kb). The significance of this difference is unknown. However, reports in the literature suggest that the 3′ UTR increases stability of the transcript and efficiency of translation^27^. Although we cannot rule out an essential role for the 5′ UTR in *Clrn1* gene rescue at this time, since the viral vector that rescued function in the KO-TgAC1 mice contains both 5′ and 3′ UTR (Fig. 6B), we strongly suspect that the 3′ UTR is critical for a robust gene rescue effect. We hypothesize that the 3′ UTR of *Clrn1* plays a critical role in post-transcriptional regulation of gene expression, by modulating mRNA localization^28–30^, stability^27^, and/or translation^31,32^, as has been shown in other eukaryotic genes.

The strategies and findings reported here could assist the basic and translational investigation of HHL linked to other genes. Recent papers mark important milestones in gene therapy and add to advances toward treatment of HHL^33–39^. These advances have led to the restoration of inner ear function to varying degrees in mouse models of HHL. Our report sheds light on the limited therapeutic window for intervention in a conventional KO mouse model, and the need to consider untranslated sequence of the target gene in gene therapy. In mouse models of syndromic hearing loss, such as those associated with USH, hair cells show signs of degeneration (disrupted or missing stereocilia and/or missing hair cells) a few days after birth. Once cellular degeneration begins, it is likely that gene therapy will not reverse this process. The intervention must occur prior to the onset of degenerative events in the cochlea, which may explain why gene therapy efforts in the field have been largely restricted to treatment of neonate mice. In the USH KO models, we surmise that prenatal delivery or a strategy to delay the onset of degenerative events will be necessary to achieve robust hearing preservation. We show that gene therapy in the progressive (KO-TgAC1) hearing loss model for USH3 prevented deafness, but the same treatment did not prevent deafness in a conventional (KO) mutant model for USH3 (Fig. 6D). In addition, robust (~4 orders of magnitude better than controls) and sustained hearing preservation is aided by the presence of UTRs flanking the *Clrn1* coding sequence; viral vectors carrying the *Clrn1* coding sequence showed only some degree of hearing preservation early on, but it was not sustained (Fig. 6D). The strategy to delay the onset of hearing loss described in our report could help widen the postnatal therapeutic window in mouse models of HHL to gauge the efficacy of the gene therapy approach. We also believe that the inclusion of UTRs in gene therapy vectors will enhance the therapeutic outcome in other mouse models of HHL and other monogenic disorders in general.

In summary, virally mediated gene delivery in a mouse model of progressive hearing loss in USH3 can 1) effectively prevent the onset of deafness associated with a *Clrn1* mutation and 2) deliver robust, long-term hearing preservation if the treatment is applied prior to the onset of the phenotype. Combined with early genetic diagnosis and family history, identifying USH3 individuals at-risk and intervening before they develop symptoms is a real possibility. Based on the results from our report and characteristics of USH3, we believe that clarin-1 is well suited for further development as a gene-therapy strategy to prevent deafness associated with USH3.

## Materials and Methods

### Mice

The transgenic mouse founder labeled TgAC1 (Transgene Atoh1-enhancer-Clrn1) was generated in the Case Transgenic and Targeting Facility under IACUC protocol number 2011–0094. The TgAC1 mice were bred with *Clrn1* homozygous knockout mice (*Clrn1* ^*KO*/*KO*^, labeled “KO”) to generate KO-TgAC1 mice. All protocols described in this report were approved by the Institutional Animal Care and Use Committee (IACUC) at Case Western Reserve University (CWRU) (protocol number 2010–0074 and 2013–0122) and at the University of California San Francisco (UCSF) (approval number AN098643–03). All procedures and animal handling complied with NIH ethics guidelines and approved protocol requirements of the IACUC at CWRU and UCSF. In this study, C57BL/6J mice were used as the wild type (WT) mice, and all the WT control material (DNA, RNA or protein) was obtained from this genetic background. All mice used in this study were maintained in the C57BL/6 J genetic background.

### Generation of transgenic line KO-TgAC1

The aim was to generate *Clrn1* ^*KO*/*KO*^ that conditionally expresses the *Clrn1* gene in hair cells from embryonic stages and down-regulate it postnatally. We generated a construct in which the *Atoh1* enhancer (GenBank: AF218258) and β-globin basal promoter (nt 86 to 133 from human ß-globin gene; GenBank: KJ480748) fused to cDNA, representing a full-length transcript of mouse *Clrn1*, including 5′ and 3′ UTRs (GenBank:NM_153385.3). The *Atoh1* enhancer element- ß-globin basal promoter construct was used previously to achieve hair cell specific expression of target genes^20,21^. Mice carrying the Transgene Atoh1-enhacer-*C* *lrn*-*1* (TgAC1) were produced by pronuclear microinjection of TgAC1 into the WT male pronucleus of fertilized eggs from WT C57BL/6J mice (http://ko.cwru.edu/services/transgenics.shtml). The scheme to generate KO-TgAC1 mice is described later (Fig. 1).

### Genotype identification

A PCR-based protocol was used to identify the TgAC1 mice. Genomic DNA was isolated as described previously^8^. The primers used for genotyping were 5′-CCCTCTCTCACACCCCATTA-3′ (KA1109) and 5′-TGAGAACCGGAAAGGCCTTGC-3′ (KA1085). The expected size of the PCR product is 1,938 base pairs (bp). *Clrn1* and *Clrn1* knockout allele were identified as described previously^8^.

### Preparation of recombinant AAV vectors

The mouse clarin-1 (*Clrn1*) cDNA with its 5′ and 3′ UTR sequences was 2,978 base pairs in length, and comprised a 5′ UTR (172 bp), the mouse *Clrn1* coding sequence (699 bp), and a large 3′ downstream UTR (2107 bp) (GenBank Accession number: NM_153384.3; Fig. 2). A construct containing only the above 5′ and 3′ UTR sequences and a unique NotI site between them was synthesized by GenScript (Piscataway, NJ). This fragment containing flanking XhoI and BamHI restriction sites was directionally cloned into an AAV vector to generate a parent plasmid devoid of transgene. The *Clrn1* coding sequence including 10 nucleotides 5′ proximal to the ATG start-codon was inserted via NotI in the above parent construct to generate “smCBA-*Clrn1*-UTR” plasmids. Transgene expression (*Clrn1* or hGFP) is driven by the ubiquitous, constitutive smCBA (small chicken β-actin) promoter. The smCBA-*Clrn1*-UTR construct was separately packaged in the AAV2 or AAV8 capsid serotypes^40^. All vectors were produced, packaged, and purified according to previously reported methods^40–42^. Viral vector titers were determined by real-time PCR. Resulting titers for AAV2-smCBA-*Clrn1*-UTR (AAV2-Clrn1-UTR) and AAV8-smCBA-*Clrn1*-UTR (AAV8-Clrn1-UTR) were 8.60 × 10^12^ and 3.41 × 10^13^ vector genomes per milliliter (vg/mL), respectively.

### Viral vector delivery

AAV2 or AAV8 were delivered to the KO-TgAC1 mice cochlea using the protocol described previously^43^. For gene therapy experiments, mice were injected at postnatal day 1–3 (P1-P3) for AAV2 or AAV8 vector delivery to the inner ear through the round window membrane (RWM). Auditory testing and cochlear histology were conducted at P21 and later. Mice were anesthetized either by hypothermia anesthesia (by placing them on ice for mice younger than P4), or by intraperitoneal injections of ketamine (100 mg/kg) and xylazine (10 mg/kg) (for older mice). Depth of anesthesia was continuously checked by deep tissue response to toe pinch. Body temperature was maintained with a heating pad and monitored with a rectal probe throughout the procedures. Animals were closely monitored for signs of distress and abnormal weight loss postoperatively. Mice of either sex were used.

### Whole mouse cochlear epithelium for GFP imaging

Cochlear epithelium was dissected from P1 or P11 *Atoh1*-GFP mice and kept in cold phosphate buffered saline (PBS) for whole mount imaging. The protocol for processing and imaging was followed as described previously^20^.

### Auditory-evoked brainstem response (ABR)

ABRs reflect the electrical responses of both the cochlear ganglion neurons and the nuclei of the central auditory pathway to sound stimulation^40,41^, and ABR thresholds refer to the lowest sound pressure level (SPL) that can generate these electrical responses. To evaluate hearing in KO-TgAC1 mice, we recorded ABR to pure tone frequencies, which represented the low (8 kHz), mid (16 kHz) and high (32 kHz) frequency ranges of the mouse cochlea. Since the *Atoh1*-enhancer is known to drive expression uniformly in all hair cells in the organ of Corti^20,21^, ABR from three characteristic frequencies along the tonotopic gradient of the cochlea should be representative of cochlear function in the KO-TgAC1 mice. Pure tone ABRs were recorded as previously described^10^.

Our goal is to determine whether a gene therapy approach can slow down, or prevent, the progression of hearing loss in the KO-TgAC1 model and, if so, whether that is a transient effect or whether that effect is sustained beyond the time point where untreated KO-TgAC1 mice normally become deaf (>P70). To determine the efficacy of gene therapy in the KO-TgAC1 mice following viral vector delivery (described later), ABR thresholds to broadband click stimuli were recorded. Thresholds to click stimulus reflect hearing function in the 4–16 kHz region of the mouse cochlea. Although a higher frequency region (16–32 kHz) of the mouse cochlea cannot be evaluated with confidence using the click stimulus, this approach is sufficient for the purposes stated above. Mice were anesthetized by an intraperitoneal injection of ketamine hydrochloride and xylazine hydrochloride as described previously^42^. After placing subdermal needle electrodes at the scalp vertex, below the pinna of the left ear and below the contralateral ear (ground), sounds were presented and ABRs were recorded in free-field conditions as previously described in a soundproofed chamber^42,43^. ABR thresholds were determined postoperatively at various time points, from 4 to 22 weeks after viral delivery. The lowest stimulus level that yielded a detectable ABR waveform was defined as the threshold, verified by visual inspection, with complementary computer analysis also defining ABR hearing thresholds for click stimuli.

### Statistical analysis

Statistical methods used to analyze ABR data from KO-TgAC1 mice (Fig. 4): ABR threshold values of both left and right ears were averaged from each mouse belonging to wild-type (*Clrn1* ^+/+^), KO (*Clrn1* ^*KO*/*KO*^) and KO-*TgAC1* (*Clrn1* ^*KO*/*KO*^; TgAC1) mice at five time points (P22, P35, P46, P55 and P70) shown in Fig. 4, using Excel (Microsoft®, Redmond, VA). The mean ABR threshold from the groups at each time point were displayed as data points (color dot/symbol) with the standard error bars on a line graph (Fig. 4). One-way ANOVA analyses were used to determine whether the differences in ABR thresholds observed between the groups were significant. *p* values of <0.05 were considered statistically significant. One-way ANOVA analysis and line graphs were done using GraphPad software.

Power of sample size used for KO-TgAC1 and gene therapy treated groups (Fig. 6D): A power analysis was performed to determine whether the sample size used in the AAV viral mediated gene therapy experiment groups compared in each cohort was statistically significant (Fig. 6D). A power analysis to determine the effective sample size in each group was done as described previously^44,45^. Since no previous data is available for gene therapy experiments in a USH3 model, a priori power analysis for sample size calculation is not possible. Therefore, it was done posteriori with the outcome of this study. A post-hoc power analysis for sample size was carried out with the observed mean difference in the ABR threshold of 38 dB SPL from P90 to P150 between the untreated (sham surgery KO-TgAC1) and treated (KO-TgAC1 -AAV-*Clrn1*-UTR) groups using the G*Power 3.1.9.2 software^45^. From the power analysis, it was estimated that to achieve a 95% chance (power) of detecting >30 dB SPL mean difference in ABR threshold between groups, an ‘n’ of 5 is necessary for each group at time points after the onset of progressive hearing loss in the untreated (KO-TgAC1) group. A student’s *t*-test was done for the power of sample size, and a *p*-value of <0.01 was considered significant.

### Scanning electron microscopy

Inner ears were excised from the head and fixed by intra-labyrinthine perfusion with 2.5% glutaraldehyde in 0.1 M sodium cacodylate buffer containing 2 mM CaCl_2_. They were immersed in the fixative for 2 h and then stored in 1/10^th^ fixative diluted with buffer until collected for further preparation. The samples were dissected in the buffer and immersed in 1% OsO_4_ in a sodium cacodylatre buffer for 1 h. Next, they were washed thoroughly and immersed in a saturated aqueous solution of sodium thiocarbohydrazide for 20 min. Again, they were washed thoroughly and immersed in 1% OsO_4_ in a sodium cacodylatre buffer for a further 2 h. The thiocarbohydrazide-osmium steps were repeated and the samples were then dehydrated in a graded series of ethanols, up to 100% ethanol dried over molecular sieve. They were placed in 100% dry ethanol in a critical point dryer, dried using liquid CO_2_ as the transitional fluid and mounted on stubs for insertion into an S4500 cold-field emission scanning electron microscope (FESEM) operated at 5 kV.

### Surgical procedures

The procedure for viral micro-injection through the cochlear round window membrane (RWM) was performed on the neonatal mouse. Mice were anesthetized as previously described and a left post-auricular approach was used to expose the tympanic bulla. Subcutaneous tissue dissection with small scissors exposed the post-auricular muscle. After retracting the adipose tissue to the posterior side of the incision, the muscles were separated to the right and left side, perpendicular to the incision, to expose the otic bulla. The glass micropipette was inserted into the RWM through the soft otic bulla, avoiding the stapedial artery. A fixed volume of the viral vector [2 µl of AAV2-GFP (2.29 × 10^12^ vg/ml) or AAV8-GFP (2.13 × 10^13^ vg/ml) or AAV2-*Clrn1*-UTR (8.6 × 10^12^ vg/ml) or AAV8-*Clrn1*-UTR (3.4 × 10^13^ vg/ml)] previously drawn into the glass micropipette was gently injected through the RWM into the scala tympani over 1 min. To allow the vector to spread throughout the cochlear duct, the glass micropipette was left in place for ~1 min after the injection. Because the hole in the RWM was extremely small, leakage of perilymph was found to be insignificant after removing the micropipette. The incision was then sealed with connective tissue, and mice were kept in an isolated warm cage until they were fully recovered from anesthesia. They were then moved back with the mother.

### Cochlear whole mount immunofluorescence

Mouse cochleae were perfused with 4% PFA in 0.1 M PBS (pH 7.4) and incubated in the fixative for 2hrs at 4 °C. The cochleae were subsequently rinsed with 0.1 M PBS three times for 10 min and then decalcified with 5% EDTA in 0.1 M PBS. The otic capsule, the lateral wall, the tectorial membrane, and Reissner’s membrane were removed in that order. The remaining organ of Corti was further dissected for a surface preparation (microdissected into individual turns), then pre-incubated for 1hr in the blocking buffer containing 0.25% Triton X-100 and 5% normal goat serum. For GFP labeling, the whole mount was then incubated with a rabbit anti-GFP antibody (Invitrogen A11122) diluted to 1:250 in 0.1 M PBS. After an overnight incubation at 4 °C, the cochlea turns were rinsed twice for 10 min with PBS and then incubated for 2hrs in goat anti-rabbit IgG conjugated to Cy2 (Jackson ImmunoResearch) diluted to 1:2000 in PBS.

## References

[1] Dror AA, Avraham KB (2009). Hearing loss: mechanisms revealed by genetics and cell biology. Annu Rev Genet.

[2] Petit C (2001). Usher syndrome: from genetics to pathogenesis. Annu Rev Genomics Hum Genet.

[3] Pakarinen L, Karjalainen S, Simola KO, Laippala P, Kaitalo H (1995). Usher’s syndrome type 3 in Finland. Laryngoscope.

[4] Ness SL (2003). Genetic homogeneity and phenotypic variability among Ashkenazi Jews with Usher syndrome type III. J Med Genet.

[5] Plantinga RF (2005). Serial audiometry and speech recognition findings in Finnish Usher syndrome type III patients. Audiol Neurootol.

[6] Joensuu T (2001). Mutations in a novel gene with transmembrane domains underlie Usher syndrome type 3. Am J Hum Genet.

[7] Adato A (2002). USH3A transcripts encode clarin-1, a four-transmembrane-domain protein with a possible role in sensory synapses. Eur J Hum Genet.

[8] Geng R (2009). Usher syndrome IIIA gene clarin-1 is essential for hair cell function and associated neural activation. Hum Mol Genet.

[9] Geller SF (2009). CLRN1 is nonessential in the mouse retina but is required for cochlear hair cell development. PLoS Genet.

[10] Geng R (2012). The mechanosensory structure of the hair cell requires clarin-1, a protein encoded by Usher syndrome III causative gene. J Neurosci.

[11] Gopal SR (2015). Zebrafish Models for the Mechanosensory Hair Cell Dysfunction in Usher Syndrome 3 Reveal That Clarin-1 Is an Essential Hair Bundle Protein. J Neurosci.

[12] Maecker HT, Todd SC, Levy S (1997). The tetraspanin superfamily: molecular facilitators. FASEB J.

[13] Hemler ME (2005). Tetraspanin functions and associated microdomains. Nat Rev Mol Cell Biol.

[14] Delaguillaumie A (2004). Tetraspanin CD82 controls the association of cholesterol-dependent microdomains with the actin cytoskeleton in T lymphocytes: relevance to co-stimulation. J Cell Sci.

[15] Tian G (2009). Clarin-1, encoded by the Usher Syndrome III causative gene, forms a membranous microdomain: possible role of clarin-1 in organizing the actin cytoskeleton. J Biol Chem.

[16] Adato A (2005). Interactions in the network of Usher syndrome type 1 proteins. Hum Mol Genet.

[17] Reiners J, Nagel-Wolfrum K, Jurgens K, Marker T, Wolfrum U (2006). Molecular basis of human Usher syndrome: deciphering the meshes of the Usher protein network provides insights into the pathomechanisms of the Usher disease. Exp Eye Res.

[18] Kremer H, van Wijk E, Marker T, Wolfrum U, Roepman R (2006). Usher syndrome: molecular links of pathogenesis, proteins and pathways. Hum Mol Genet.

[19] Boeda B (2002). Myosin VIIa, harmonin and cadherin 23, three Usher I gene products that cooperate to shape the sensory hair cell bundle. EMBO J.

[20] Lumpkin EA (2003). Math1-driven GFP expression in the developing nervous system of transgenic mice. Gene Expr Patterns.

[21] Chen P, Johnson JE, Zoghbi HY, Segil N (2002). The role of Math1 in inner ear development: Uncoupling the establishment of the sensory primordium from hair cell fate determination. Development.

[22] Abdolazimi Y, Stojanova Z, Segil N (2016). Selection of cell fate in the organ of Corti involves the integration of Hes/Hey signaling at the Atoh1 promoter. Development.

[23] Jero J (2001). Cochlear gene delivery through an intact round window membrane in mouse. Hum Gene Ther.

[24] Kilpatrick LA (2011). Adeno-associated virus-mediated gene delivery into the scala media of the normal and deafened adult mouse ear. Gene Ther.

[25] Zallocchi M (2012). Role for a novel Usher protein complex in hair cell synaptic maturation. PLoS One.

[26] Keller, J. M., Neely, H. R., Latoche, J. R. & Noben-Trauth, K. High-frequency sensorineural hearing loss and its underlying genetics (Hfhl1 and Hfhl2) in NIH Swiss mice. *J Assoc Res Otolaryngol* **12**, 617–31 (2011). 10.1007/s10162-011-0270-7 PMC3173551 21594677

[27] Bashirullah A, Cooperstock RL, Lipshitz HD (2001). Spatial and temporal control of RNA stability. Proc Natl Acad Sci USA.

[28] Du TG, Schmid M, Jansen RP (2007). Why cells move messages: the biological functions of mRNA localization. Semin Cell Dev Biol.

[29] Jansen RP (2001). mRNA localization: message on the move. Nat Rev Mol Cell Biol.

[30] Palacios IM, St Johnston D (2001). Getting the message across: the intracellular localization of mRNAs in higher eukaryotes. Annu Rev Cell Dev Biol.

[31] Kuersten S, Goodwin EB (2003). The power of the 3′ UTR: translational control and development. Nat Rev Genet.

[32] Mazumder B, Seshadri V, Fox PL (2003). Translational control by the 3′-UTR: the ends specify the means. Trends Biochem Sci.

[33] Lentz JJ (2013). Rescue of hearing and vestibular function by antisense oligonucleotides in a mouse model of human deafness. Nat Med.

[34] Yu Q (2014). Virally expressed connexin26 restores gap junction function in the cochlea of conditional Gjb2 knockout mice. Gene Ther.

[35] Askew C (2015). Tmc gene therapy restores auditory function in deaf mice. Sci Transl Med.

[36] Pan B (2017). Gene therapy restores auditory and vestibular function in a mouse model of Usher syndrome type 1c. Nat Biotechnol.

[37] Isgrig K (2017). Gene Therapy Restores Balance and Auditory Functions in a Mouse Model of Usher Syndrome. Mol Ther.

[38] Gyorgy B (2017). Rescue of Hearing by Gene Delivery to Inner-Ear Hair Cells Using Exosome-Associated AAV. Mol Ther.

[39] Landegger LD (2017). A synthetic AAV vector enables safe and efficient gene transfer to the mammalian inner ear. Nat Biotechnol.

[40] Petrs-Silva H (2009). High-efficiency transduction of the mouse retina by tyrosine-mutant AAV serotype vectors. Mol Ther.

[41] Zolotukhin S (1999). Recombinant adeno-associated virus purification using novel methods improves infectious titer and yield. Gene Ther.

[42] Jacobson SG (2006). Safety of recombinant adeno-associated virus type 2-RPE65 vector delivered by ocular subretinal injection. Mol Ther.

[43] Akil O (2012). Restoration of hearing in the VGLUT3 knockout mouse using virally mediated gene therapy. Neuron.

[44] Dupont WD, Plummer WD (1990). Power and sample size calculations. A review and computer program. Control Clin Trials.

[45] Faul F, Erdfelder E, Buchner A, Lang AG (2009). Statistical power analyses using G*Power 3.1: tests for correlation and regression analyses. Behav Res Methods.

